# Perceptions of South African Emerging Adult FET College Students on Sexual Practices in Relation to Religion

**DOI:** 10.1007/s10943-016-0312-x

**Published:** 2016-10-11

**Authors:** Colleen Gail Moodley

**Affiliations:** Department of Mechanical Engineering: Mechatronics, Faculty of Engineering, Cape Peninsula University of Technology, PO Box 1906, Symphony Way, Bellville, Cape Town, 7535 South Africa

**Keywords:** Religion, Sexual practices, Sexual decision-making, Emerging adulthood, College students

## Abstract

HIV and AIDS are rapidly spreading amongst the world’s 15- to 24-year age group, particularly in sub-Saharan Africa. Despite vigorous government interventions and campaigns, 10 % of South African youth in the age cohort 15–24 are infected with HIV and AIDS. Furthermore, for the first time in history the world has its largest number of individuals under the age of 30 years. Researchers are desperately seeking a solution and have found religion to play an important role in moderating risky sexual behaviour amongst youth. This exploratory qualitative study aims to increase our understanding of emerging adult Further Education and Training (FET) students’ perceptions of the role of religion and religious beliefs in their sexual decision-making and practices. The qualitative data emerged from five focus group discussions, each consisting of 12 heterosexual emerging adult FET college students aged 18–24 years, selected using random sampling. Participants were representative of all the major South African racial groups (Blacks, Whites, Coloured and Indians) as well as different religious and cultural groupings. Secularisation theory was used as a theoretical framework for this study. These focus group discussions revealed the following themes: Theme 1—religious institutions need to embrace change in order to become effective social agents of change. Theme 2—a need for open discussion and communication concerning current issues related to young people’s sexual health (by religious institutions/religious leaders). Theme 3—perceptions of religion’s negative sanctions towards sexual behaviour. Theme 4—religious leaders’ indifference and abdication of responsibility to the problems that youth face. Theme 5—religion and condom-related beliefs. Theme 6—perceptions of religious leaders as role models. Theme 7—emerging adults general concern for the moral decay of society. Theme 8—perceptions of whether religion has an influence on young people’s sexual decision-making and practices.

## Introduction

### HIV and AIDS Trends for the Age Cohort 15–24 Years (Emerging Adults)

The world is experiencing a unique phenomenon, for the first time there are the largest number of people under the age of 30 years, 3.6 billion (Madsen et al. 2010). South African studies show that, despite various government initiatives in countering the spread of AIDS, 10 % of South African 15- to 24-year-olds are HIV positive (NSP 2007–2011, 2007). HIV trends indicate that sub-Saharan African youth are the most affected by HIV and AIDS (Bankole et al. 2007; UNAIDS 2013).

Currently, the United Nations and AIDS (UNAIDS) report on the global AIDS epidemic for 2013 (UNAIDS 2013) reflects positive trends for HIV in sub-Saharan Africa for the age cohort 15–24 years. Knowledge of the prevention of HIV has increased; there has been a decline in the number of 15- to 24-year-olds who have engaged in sexual intercourse before the age of 15 years; condom use has increased amongst those with multiple sexual partners, with an increase in the number of those individuals who have had HIV testing and learned their HIV status. Between 2001 and 2011, prevalence of HIV-a proxy indicator of new HIV infections declined by nearly 27 % amongst young people aged 15–24 globally (UNAIDS 2012).

However, despite these positive indicators, new AIDS infections are occurring rapidly in the age group 15–24 years (UNAIDS 2009). And indicators for the African continent show a significant increase in the number of sexual partners in the following countries: South Africa, Uganda, Congo, Burkina Faso Gabon, Guyana, Côte d’Ivoire, Ethiopia, Rwanda, the United Republic of Tanzania and Zimbabwe, with a decline in the use of condoms in Côte d’Ivoire, Niger, Senegal and Uganda (UNAIDS 2013).

This desperate situation has prompted scholars such as Madsen et al. 2010; Mantell et al. 2011; Marston and King 2006; Mash and Mash 2012; Mutinta and Govender 2012; Sinha et al. 2007; Sinha et al. 2006 to rethink current means of curbing the HIV epidemic and to focus particularly on the emerging adult age cohort as a means of approaching the rampant spread of HIV amongst young people. Taking cognisance of this, religion has been touted as a means of contributing towards pro-social behaviour change in this age cohort, including sexual practices (Madsen et al. 2010; Mantell et al. 2011; Marston and King 2006; Mash and Mash 2012; Mutinta and Govender 2012; Sinha et al. 2006, 2007; UNAIDS 2013).

## Emerging Adulthood as a Developmental Phase

Arnett (2000) coined the term *emerging adulthood*, referring to the age group 18–24 years. He defines emerging adulthood as a developmental phase distinct from adolescence where identity development is crucial and exploration in areas such as work, love and ideology is central. The focus therefore tends to be on self-development and developing independence. Religion and sexuality are also two key areas for the emerging adult development phase and may be especially impressionable during this period of maturation (Arnett 2000). Religious practices may have been prescribed by family while living at home; however, individuals entering college (or university) may have more opportunities to re-examine different religions and beliefs (Arnett 2000). Emerging adulthood is thus a period of exploration in the areas of religion and religiosity (Arnett 2000). The development process therefore includes that most emerging adults form a distinctive set of beliefs about religious issues. Several studies have indicated that deciding on one’s own beliefs and values is one of the criteria young people view as most important in becoming an adult (Arnett 1997; Greene et al. 1992).

### Emerging Adult Students and Sexual Behaviour

This study targets students for numerous reasons. Scholly et al. (2005) posit that college students represent an important population for studying and understanding factors that influence sexual risk. Risky sexual behaviour can compromise students’ academic success and may result in life-altering and life-threatening consequences such as sexually transmitted infections (STIs), HIV and unplanned pregnancies. Pluhar et al. (2003) postulate that data collected while young people are in college are important as it is at a time when sexual values are being further explored and concretised.

### South African Emerging Adult Student Sexual Behaviour

Lengwe (2009) focused on investigating students’ sexual risk behaviour, in line with the *Scrutinise Campus Campaign* prevention programme held on campuses at various South African universities. His study found a dramatic increase in HIV prevalence amongst students from their late teens to early 20s and even more so after 25 years. Moodley’s (2010) study amongst emerging adult, Further Education and Training (FET) college students in the Western Cape, found that environmental factors such as culture, poverty, use of drugs and religion have an effect on emerging adult students’ sexual decision-making. Recent South African research conducted by Mutinta and Govender (2012) on emerging adult students’ sexual risk behaviour and HIV prevention at the University of KwaZulu-Natal concurs with these findings. It was established that the risk of HIV infection is initiated by contextual factors that include socio-environmental factors that greatly influence students’ sexual risk-taking behaviour (Mutinta and Govender 2012).

Since the most common age cohort, attending Further Education and Training (FET) and Higher Education and Training (HET) institutions, are emerging adults, individuals aged 18–24 years (HEAIDS 2010), the current study’s population falls within this age cohort. Furthermore, it should be noted that there is a paucity of studies in particular the FET college sector investigating areas for intervention and sexual health promotion. Ferreira (2002) found that young people at FET colleges have insufficient knowledge, confidence and life skills to negotiate sexual issues, contraception, prevention of STIs and HIV and AIDS. The baseline survey and needs assessment of FET colleges, conducted by the Planned Parenthood association of South Africa (PPASA 2004) amongst six FET colleges in the Western Cape, support Ferreira’s findings. Despite the inclusion of life skills into the FET college curricula with new programmes such as NCV, FET colleges offer very little support in terms of health promotion programmes (Western Cape Education Department [WCED] 2006).

While numerous studies have found religion to be an important influence on moderating risky sexual behaviour amongst youth (Fehring et al. 1998; Mantell et al. 2011; Regnerus 2003; Sinha et al. 2006, 2007), few studies have explored the content of emerging adults’ religious beliefs in relation to their sexual decision-making and practices (Arnett and Jensen 2002).

Thus, the objective of this paper is to explore the views and experiences of particularly Further Education and Training (FET) college students in Cape Town, South Africa, concerning the role of religion as a factor affecting their sexual behaviour and in turn their sexual health. This study therefore seeks to fill this gap by shedding light on the role of religion in the sexual practices of emerging adult (FET) college students.

## Literature Review

### The Conceptualisation of Religion

There are various definitions of the concept—religion. For the purposes of this study, Pargament’s (1997) definition of religion will be used. He defines religion as a multidimensional construct that includes both institutional religious expressions, such as dogma and ritual, and personal religious expressions, such as feelings of spirituality, beliefs about the sacred and religious practices. Various sociologists have concurred and affirmed the important role of religion in society. For example, Marx (1964) asserted that religion is instrumental in serving ruling elites by legitimising the status quo and diverting the masses attention from the inequities in society. Berger (1967) viewed religion as a social construction, where everyday life was placed under a “sacred canopy” of meaning.

### Emerging Adulthood and Religion

Two areas of development during emerging adulthood that are especially impressionable are religiosity and sexuality (Arnett 2000). Barry and Nelson (2005) support this view by contending that emerging adulthood is defined by heightened risk-taking behaviour and self-exploration of various domains, including spirituality. Beaudoin (1998) posits that it is common for this age group (late teens and early 20s) which he refers to as “Generation X” to be sceptical about the significance of religious institutions and to remain open to changing their beliefs rather than arriving on a fixed set of beliefs. In addition, this age cohort also has a tendency to emphasise personal experience before religious authorities as a source of religious beliefs. Tiendrebeogo and Buykx (2004, p. 53) analysed a literature review compiled by The Royal Tropical Institute, Netherlands, where the activities of faith-based organisations in responding to HIV and AIDS in sub-Saharan Africa were examined. These researchers asserted that “more research is needed to document the influence of religion on behaviour change and to assess the effects and processes of faith based organisations”. Puffer et al. (2012) and Mash and Mash (2012) concur. In light of the above views, this study aims to contribute towards further elucidation of the role of religion in behaviour change, specifically with regard to sexual practices.

### The Role of Religion in Sexual Decision-Making

Numerous studies (Byers et al. 2009; Mantell et al. 2011; Puffer et al. 2012) have found religion to be an important influence on moderating risky behaviour amongst youth. For example, Puffer et al. (2012) found evidence that religious coping may influence how some adolescents in sub-Saharan Africa respond to poverty and decisions about sexual behaviour. Adolescents reported praying to God to collaborate with them to avoid risk behaviour, to meet their economic needs, and to protect their health. In this manner, religious coping seemed protective. Other studies report that participants who were sexually abstinent (mostly defined as never having engaged in vaginal intercourse) had more conservative values and sexual attitudes, few sexual experiences of any kind and greater religious involvement (Byers et al. 2009; Mantell et al. 2011). Regenerus’ (2003) review of research concerning adolescent risk behaviours, including the use of alcohol and drugs, and engaging in sexual intercourse, found that religion seemed to have a protective influence and also distinguished (to a greater or lesser extent) between those adolescents that participated in such behaviours and those that did not. Sinha et al. (2007) found that the perceived importance of religion was significantly associated with eight of ten youth risk behaviour variables, including sexual activity. Fehring et al. (1998) conducted studies on the effects of religion amongst youth (aged 17–22 years) and found that a high rating of significance of faith (coupled with orthodox beliefs, and involvement in organised religious activities) predicted less permissive sexual attitudes.

Gender may be a mediating factor in the role that religion plays in sexual decision-making and sexual practices. For example, Sinha et al. (2006) found that Muslim and Hindu women were less likely to have had sex compared to those who reported no religious association. They also found that for young men, religion was not significantly associated with having had sex. In addition, for both sexes, religion per se did not seem to influence contraception use. However, regular attendance of religious ceremonies was associated with a lower risk of having had sex and having unprotected sex amongst young men but not young women. Adamczyk and Hayes (2012) found similar results. Their study focused on analysing behaviours rather than attitudes, and their findings indicate that Hindus and Muslims are significantly less likely to report having had premarital sex than Christians and Jews.

Sub-Saharan African studies on the effects of religion on sexual practices have largely focused on contraceptive use intentions and to protect oneself against out-of-wedlock pregnancies. However, a Zambian study found that the effect of religious affiliation on the risk of HIV infection may be minimal. If the risk-reducing effect of affiliation to conservative groups is only temporary (i.e. affiliation delays sexual initiation, but reduces the likelihood of condom use when sexual initiation takes place), the overall protective effect of belonging to conservative religious groups on the risk of HIV infection may be minimal (Agha et al. 2006).

Caution should, however, be taken when making conclusions about the role of religion and religious values in sexual practices and sexual decision-making. Donelson (1999) advises that because “religiosity” tends to be conceptualised and measured inconsistently across studies, it therefore becomes difficult to make sound conclusions concerning the precise nature of the link between religiosity and adolescent well-being.

### Theoretical Framework: Secularisation Theory

Secularisation theory has been used to explain the relationship between religion and sexual behaviour in this study. This theory suggests that modern society’s dependence on reason has resulted in an uncertainty concerning the role of religion and purports the limited influence of religion on daily life at an individual, social and institutional level (Berger 1967; Sommerville 1998). Secularisation theory also predicts the eventual demise of religious involvement in modern secular life. Secularisation theory informed the analysis of data in this study, and thus the theory is examined in more detail.

### A Brief Overview of Secularisation Theory

Pickel (2011) posits that there is no single secularisation theory. The varied nature of secularisation theory suggests a multidimensional concept since there is no apparent unified theory of secularisation (Bruce 2002; Spickard 2003). Rather, the term refers to numerous ideas that all relate to one key relationship—the fundamental tension between modernisation and religion. Secularisation theory is therefore varied in terms of various aspects such as its definitions, interpretations, assumptions about the past, present and future social scenarios, measurement approaches and levels of analysis. For example, Shiner (1967) included six versions of secularisation used by sociologists in empirical work. These are (1) the decline of religion—where religious, doctrines, symbols and institutions lose social significance; (2) conformity with the world—where religious organisations focus on the goals of “this world” rather than the “next”/spiritual; (3) disengagement—where religious organisations lose some functions to other institutions, thereby losing significance in moral and political terms; (4) transposition of religious beliefs and institutions—where issues that were previously regarded as steeped in divine power are considered “human creations”; (5) desacralisation of the world—rational and scientific explanations override religious faith; and (6) from sacred to secular society—religion moves from its central position to a “market” of other possible philosophies.

The varied nature of secularisation theory has been the source of much criticism. Despite these criticisms, Pickel (2011) highlights that critics often fail to see the common focus of the different approaches of secularisation theory—the tension between modernisation and the development of religion. In fact, the varied nature of secularisation theory is its main advantage in terms of its range and multidimensionality explanatory approaches (Bruce 2002; Dobbelaere 2002; Chaves 1994).

### Reasons for the Spread of Secularisation

Pickel (2011) suggests that rationalisation and modernisation result in the disintegration of the transfer of religious knowledge and traditions across generations. An increase in standards of living in modern societies correlates with a decrease in the importance of religion. Modern welfare states in particular are characterised by high levels of secularisation (Norris and Inglehart 2004). These factors are accompanied by increasing bureaucratisation, urbanisation and democratisation, resulting in the collapse of hierarchies important to religious organisations (Pickel 2011).

Crabtree (2012) considers multiculturalism as the reason for secularisation. Communities that once shared similar religious beliefs and morality are fast vanishing. Furthermore, individuals are exposed to a wider range of beliefs provided by various modern technologies that spread information of other cultures and religions. Crabtree (2012) blames the Internet for secularisation. He asserts that previously, big and established religious organisations monopolised and dominated national broadcast media because of the access they had to more finances, the national media and greater output power. Currently, technological communication such as the Internet allows dissenting voices of individuals and sects to freely present their individual points of view. Crabtree also considers human rights as a reason for secularisation. Religious groups and individuals advocate human rights laws that may be in direct conflict with religious teachings (e.g. the legalisation in South Africa of gay marriages and abortions, the use of contraception by Catholics despite institutional papal sanction).

Secularisation is often perceived as a deliberate political plan, rather than a spontaneous sociocultural development (Froese 2009; Smith 2003a). Studies identify the central role played by religious state institutions as the source of variation in secularisation across societies. Political mobilisation driven by religion is frequently used by political elites to either reduce the public role of religion (institutional secularisation) or increase state control in religious organisations. This often incites conflict (Vorster 2007).

### Secularisation’s Influence in Society

Secularisation affects society on various levels. For example, Dobbelaere (2002) identified three levels of social influence, namely: societal secularisation, organisational secularisation and individual secularisation. Casanova’s (1994) three levels of secularisation are loss of personal religiosity, growing functional differentiation, marked by increased separation of church and state, and the privatisation of religion. Alternatively, Bruce (2002) presents three aspects of secularisation: the disintegration of faith because of the decline in religious attendance of those who consider religion to be significant, decline in social significance of religion in people’s lives and in the public sphere, and the depreciation of importance of religion concerning religious organisations in society. These examples highlight the challenges as well as the potential, in using secularisation theory to explain the relationship between religion and society.

Martin (2011) posits that the process of secularism manifests itself differently in different places. He provides numerous examples of specific European histories linked to particular types of secularisation. Furthermore, Martin’s (2011) views infer possible differences between Eurocentric and African processes of secularisation. Since this study focuses on emerging adults in an African context, a brief discussion about secularisation in Africa follows.

### Secularisation in Africa

van den Toren (2003) names social change as key to secularisation in Africa. Key social changes are urbanisation, the fragmentation of life and society, globalisation and religious pluralism. Despite being the least urbanised continent, Africa is experiencing rapid urbanisation, as vast numbers of rural dwellers migrate to urban centres (van den Toren 2003). Displacement and fragmentation occur because of the impersonal character of urbanised life. Consequently, religious convictions are often relegated to the private sphere. Religion’s importance is reduced, making it obsolete and creating religious indifference. Additionally, rapid globalisation of African societies results in the adoption of capitalistic economic interests and Western secular values, promoted by media. Globalisation is engulfing a religiously *pluralist* world promoting the ideology that no particular religious value system should be followed. Thus, consumerism and capitalism now replace religious values becoming the dominant value systems in society (van den Toren 2003).

## Methodology

### Population and Sampling

The FET sector is a diverse and an essential part of the education and training system in South Africa since it provides for much needed skills required in a growing economy. The sector provides vocational or occupational specific training meaning that students receive education and training focusing on a specific range of jobs or employment potential. Under certain conditions, students may continue their studies at a higher level, in the same field at Universities of Technology. Although FET forms part of higher education, it is larger than the Higher Education and Training (HET) sector in terms of the number of student enrolments and total expenditure. It is also unique as it provides training over a large age range from 16 to 60 years and older, which differs from the traditional HET sector (universities) where most students commence their studies at age 18/19 years. Extensive government funding, for the FET’s new National Vocation Certificate (NCV) courses (commenced January 2007), has resulted in larger numbers of younger students at FET institutions.

The FET sector in the Western Cape comprises of 5 colleges (WCED 2006). It was deemed appropriate to select the college with the largest number of students. The selected FET college consists of eight campuses with approximately 5000 students in the 18- to 24-year age group. Convenience sampling was used to select participants for this study. Students across all campuses, who were in the age cohort, 18–24 years, were invited to participate in this study. A total of 48 students participated in the study. The sample consisted of 20 males and 28 females. The sample was randomly selected from the five campuses and representative of the major South African religions (Christianity, Islam, Hinduism, African Traditional Religion and Judaism) and racial groups (Blacks, Whites, Coloured and Indians).

### Research Design and Data Collection

A qualitative approach was used employing a case study method to capture the views of FET college students concerning the role and influence of religion on their sexual behaviour. According to Gall, Borg and Gall (1996:545) case study in qualitative research is defined as “the in-depth study of the instances of a phenomenon in its natural context and from the perspective of the participants involved in the phenomenon”. Four focus group discussions were held with 12 participants per group, aged 18–24 years. Participation in these focus groups discussions was voluntary.

A semi-structured interview schedule compiled by the researcher was used to generate discussion on the influence of religion on sexual behaviour. The questionnaire posed questions relating to the following: whether students had engaged in sexual behaviour, whether these sexual practices were unprotected, whether religion had an influence on their sexual decision-making and on society as a whole.

The focus group discussions took place in classrooms at the various campuses chosen for their location, guarantee of privacy, where interruptions and noise were reduced and comfortable seating was available. No interruptions occurred during the discussions.

### Data Analysis

Focus group discussions were recorded, audio-taped and transcribed verbatim.

Content analysis, using Baptiste’s model (2001), was used to analyse the data. Baptiste posits that (regardless of the methodological or disciplinary orientation) qualitative data analysis involves four interrelated phases: defining the analysis, classifying data, making connections between data and conveying the message(s).

This model provides a user-friendly explanation and structure for novice researchers and involves four phases, namely: (1) analysis of the data, (2) reading through the various scripts and selecting the detail and its classification, and labelling through the use of numbers phrases and tags into various themes, (3) summarising the concepts, (4) writing the report.

### Trustworthiness

Data verification was done using Krefting’s guidelines and the Guba model in Krefting (1991) to ensure trustworthiness. Guba’s model is based on the four aspects of trustworthiness, namely: truth, value, applicability, consistency and neutrality.

Truth value was applied by using audio recordings during the data collection process. Thus, the researcher could re-examine the data to ensure that participant accounts were authentic. Themes generated in the study were clarified through saturation of participant reports. Participants provided comments on the accuracy of the findings. Consistency/neutrality was applied by ensuring that the research process was transparent and clearly described, i.e. the outline, development of methodology and reporting of the findings of the study. The themes emanating from the study were open for discussion and could be contested by the researcher’s peers who are seasoned qualitative researchers, whereafter consensus was reached. Applicability was applied when the emerging adult participants provided in-depth details of the experiences and perceptions of their sexual behaviour. These rich accounts facilitated the evaluation of the study conclusions.

### Ethical Considerations

Ethical clearance was obtained from the Chief Executive Officer of the FET College and the Western Cape Education Department (WCED). Informed written consent was obtained from students whose participation was voluntary. Students were assured of confidentiality of the study and that they could withdraw from the study at any time. Access to psychological services was made available to students due to the sensitive nature of the focus group discussions.

## Findings and Discussion of Findings

Content analysis of the transcripts of the focus groups (which was heterogeneous in terms of culture, gender and religion) yielded the following themes: Theme 1—religious institutions need to embrace change in order to become effective social agents of change. Theme 2—a need for open discussion and communication concerning current issues related to young people’s sexual health (by religious institutions/religious leaders). Theme 3—perceptions of religion’s negative sanctions towards sexual behaviour. Theme 4—religious leaders’ indifference and abdication of responsibility to the problems that youth face. Theme 5—religion and condom-related beliefs. Theme 6—perceptions of religious leaders as role models. Theme 7—emerging adults general concern for the moral decay of society. Theme 8—perceptions of whether religion has an influence on young people’s sexual decision-making and practices. A report and discussion of these themes follows.

### Theme 1: Religious Institutions Need to Embrace Change in Order to Become Effective Social Agents of Change

Participants questioned the relevance and function of religious institutions in modern society as effective agents of social change. A male Christian student demonstrated this view as follows:

*I think religion has lost the plot in trying to tell people what to do, because they (*religious institutions) *haven’t changed with the times. They’re still stuck in the old times and they’re falling behind. If they don’t change then what is their purpose anyway? That is why so many churches are closing their doors* (losing members and having to close down)… *people, especially young people… don’t want to go to church anymore.*

This view suggests that students are sceptical concerning the function of religious institutions, as these institutions are considered outdated. The majority of the students agreed that societal perceptions concerning the influence of religion on particularly sexual behaviour had changed. They referred to the “old times” as a time when religion had been perceived as having had a moderating influence on sexual behaviour, with religious mores playing a regulatory role sexual practices.

A Christian female student summarised this as follows:

*In the “old times”* (past*) couples would* get *married, and then have sex, no abortions. If this happens* (having premarital sex or abortions) *you would not be allowed in the church (Place of worship) anymore…it would be unacceptable. A disgrace!* (Others agreed).

A Hindu female student added that

*Living or sleeping together* (having sexual intercourse) *like people do today would never have been accepted*…*and still isn’t for many… Marriages was arranged in the old days…some still are… and you would get to know* (have intercourse for the first time) *your husband on your wedding night*…

Participants felt that the messages conveyed in religious institutions should become more modern in content and therefore more appealing to a younger audience. This is displayed in the following excerpt:

A female Christian student stated the following

*In church their focus is only on changing the person. So now, I think that they must start making their sermons more interesting. Changing their sermons with the times… It’s boring in church…same old story over and over again.* (Student shrugs shoulders and throws the hands up in disillusionment). Others agree

From participant responses, it can be deduced that students felt that religion lacked the ability to effect sexual behaviour change and was seen as lacking allure and credibility. The literature concurs with this view. Leclerc-Madladla (2005) found that discarding religion in pursuit of modernity makes students susceptible to HIV infection. Wallace and Forman (1998) suggest that a partnership should be encouraged between health professionals, religious professionals and religious institutions to change and improve their perceptions of young people’s sexual health behaviours.

Concerning the role that religion and religious institutions could play concerning sexual health, Puffer et al. (2012) advise that adolescents in sub-Saharan Africa may benefit from HIV prevention interventions that incorporate their use of religious coping. Aguwa (2010) argues that religious organisations have access to young people and families and play an integral role in influencing society. South African faith-based organisations are central to community life, holding positions of trust, which affords them extensive influence in the rural and remote areas of the country. These organisations have frequent opportunities to interact with their congregations and communities and have the ability to influence social norms and behaviours through moral teachings. These organisations therefore have the ability to bring about real change as they are enmeshed in people’s real problems (Keikelame et al. 2010). HIV programmes involving young people where their beliefs and life patterns are respected have a greater chance of success than programmes that do not prioritise such a holistic approach (UNAIDS 2013). Therefore, Ricardo et al. (2010) advise that it is crucial for churches to engage with young people on issues of sex and sexuality before their behaviour patterns are established. Recent South African research has found that an HIV church programme on prevention has had a positive effect on prevention amongst youth groups (Mash and Mash 2012).

## Theme 2: A Need for Open Discussion and Communication Concerning Current Issues Related to Young People’s Sexual Health (by Religious Institutions/Religious Leaders)

Participants indicated that there was a need for open communication and discussion on issues related to young peoples’ sexual health in religious institutions.

A Christian student commented as follows:

*I think that they* (religious leaders) *should have discussions like this* (the focus group discussion on sexual health that was underway) *with their youth*…*but they should get in young people to talk to the youth*… *like somebody their own age that they can talk with. And with both males and females.*

Another Christian student stated the following:

*They should at least have those Aids people* (people who are trained in the field of HIV and AIDS) *come in once in a while to speak to us. These church leaders shouldn’t preach to us ‘you people mustn’t do It’* (have sex) *because young people will do just that, young people are doing it* (having sex)*. They must leave it up to the individual to decide if they want to do* (have sex) *it or not.*

Participants also indicated that though religious youth leaders were of the same age they were not discussing issues related to young people’s sexual behaviour.

*Yes, we have youth leaders our age but they don’t communicate with us about sex… These people are staunch in their faith and they won’t preach things that are different from what the church preaches. Their message is just abstain*… *it’s* (to engage in sexual activity) *a sin. So maybe, the church or religious institution has to change,*… *to say maybe, use condoms and wait, maybe if the right person at the right time comes along. If they do this then maybe young people will listen.*

A Xhosa male student stated that discussing sexual behaviour, regardless if one was the same age (discussion of sex with peers) as well as discussions with the elders or religious leaders was a taboo. *Imagine us sitting and talking about sex to them* (elders or religious leaders)

… *Ooh miss, that would never happen in our culture. We don’t talk about drugs and sex and stuff like that, its private we don’t talk about that*… *If you talk about sex it’s like you’re interested in it.*

Some participants disagreed with the above and felt that having religious leaders speaking to youth about current contentious issues relating to sexual behaviour would not necessarily change the views or moral behaviour of young people.

A Christian student said the following:

…*these religious people, priests,*… *imams can talk,*… *preach,*… *tell us what to do and what not to do*… *it depends on us,* (the decision to engage in unprotected sexual intercourse with multiple partners), *not them*. (Others agreed).

Participants felt strongly that religious leaders should receive training in order to facilitate conveying accurate current information to youth.

The following excerpt indicates this:

*The church should play a bigger role. There’s some pastors and priests who don’t wanna speak about HIV in their churches. They should get more training about these issues.* (All students agreed)

A Muslim student added:

*Actually all religious leaders need to be educated about how to deal with young people and the things that they* (youth) *have to go through.*

The male Muslim student further added:

*There is really a big need to educate our priests and imams*… *they are still in the dark ages …*

From the student discussions, it can be inferred that there is a real need for religious institutions and their leaders to provide information that is relevant, accurate and current. Furthermore, this information should be provided by individuals who are forthright, preferably in their age cohort, and who are able to understand problems and situations young people face. Participants noted that a change in perspective was required as there is a perception that religious leaders or elders of the community discourage open discussion and are apprehensive to address sexual health issues as this was perceived as encouraging sexual activity. Some participants felt that forthright discussions would not necessitate change in behaviour as decisions to engage in sexual behaviour are a personal moral decision over which religious leaders had no influence. However, participants proposed a possible solution to aid in the facilitation of open communication on current sexual health issues—they suggested training religious leaders (and those concerned with youth) on issues affecting young peoples’ sexual health. Various researchers concur with this view. For example, research conducted by Keikelame et al. (2010) yielded similar results. The current study’s findings further concur with Keikelame et al. (2010) concerning the view that South African clergy were perceived as lacking the knowledge and skills to deal appropriately with issues relating to HIV and AIDS, including the sexual transmission of HIV. Therefore, insufficient knowledge and social skills could add to the preaching of conflicting and confusing messages and ultimately to the unintentional promotion of HIV and AIDS stigma and discrimination. They further found that South African faith-based leaders lack the language and social skills to communicate about issues related to sex and sexuality. Findings by Boonstra (2011) show that religious leaders felt that talking about sex and condom use would legitimise young people’s sexual promiscuity. However, Paterson (2009) cautions that reluctance of religious leaders to talk about issues relating to sexual issues could unwittingly encourage young people to seek sexual direction from inappropriate sources such as pornography.

Possible reasons for the silence of some religious institutions concerning sexuality and HIV are that intercourse is considered the main conduit through which HIV is transmitted (Smith 2003b). Further empirical evidence shows that religious institutions have received considerable criticism related to their involvement in HIV prevention from HIV activists and support groups concerning the spreading of inaccurate HIV information to young people (Eriksson et al. 2011).

### Theme 3: Perceptions of Religion’s Negative Sanctions Towards Sexual Behaviour

Participants indicated that they perceived religious leaders and institutions as adopting an inflexible stance concerning young people’s sexual behaviour

A Christian male student stated:

*These people (religious leaders) are staunch*… *they won’t preach things that are different from what the church preaches*… *No sex before marriage.*

Some participants indicated that regardless of one’s religious affiliation, religion should not be used to judge young people’s behaviour.

A Hindu female responded to the issue of sex before marriage as follows:

*When it comes to religion, it doesn’t matter what religion you belong to you*…*as a leader and an ordinary person*…*you are not allowed to judge others.*

From participant responses, it can be deduced that participants considered religious leaders and religious institutions as rigid and judgemental in their beliefs. Young people were perceived as immoral and wayward. Participants felt that the rigid stance of religious leaders on sexual practices and moral behaviour needed to change. And because of their inflexibility and reproachful manner they are not gaining support from young people. Some participants felt that religious leaders should be more flexible in their approach to the issue of sexual behaviour and less judgmental.

The literature concurs with the views of participants in the current study. For example, Horn (2010) found that conservative religious institutions often promulgate rigid norms of sexual identity which is supported by an ideological stance that homosexuality is sinful. Burchardt (2011) found Pentecostal religious leaders were inclined to emphasise the preaching sexual abstinence, morals and fidelity amongst their members. Greeley (1991) found that individuals with high religiosity had a greater propensity to blame and judge others. As Gordon and Mwale (2006: 71–72) state: “Faith-based organisations have always struggled with how to marry their moral mission and the need to protect health and life, given the reality of people’s sexual lives”. Furthermore, concerning the adapting role of religion, the researcher concurs with Aguwa (2010) that a realistic, feasible option should be found that effectively addresses Africa’s and, in particular, South Africa’s cultural, social, economic and health issues. However, it is cautioned that if religious organisations and their leaders persist in maintaining rigid conservative views they may be considered as being “out of touch with reality” and a stumbling block to change (Aguwa 2010; Mantell et al. 2011).

### Theme 4: Religious Leaders’ Indifference and Abdication of Responsibility to the Problems that Youth Face is Displayed as Follows

A Xhosa male student who had lived in rural Eastern Cape referred to traditional religious leaders as lacking the ability to solve youth’s problems

*Many people go to sangoma’s,*… *No sangoma can hold the answers for young people… they don’t give you answers anyway…*

One Muslim student summarised the attitude of these religious leaders as follows:

*They* (religious leaders) *are not sending any messages, they don’t know what to say,*…*They’ve given up.*

Based on participant responses, it can be inferred that students view religious institutions and their leaders as having given up on the moral behaviour of young people. They are unable to provide solutions to young people’s problems via religion and have become apathetic towards the situation concerning young people’s sexual health. They thus seem to have absolved themselves of responsibility. Findings in a study on churches’ attitude to premarital sex conducted by Burchardt (2011) confirm this apathy. He found that the teachings of the Pentecostal churches of South Africa ban premarital sex and discussion thereof. However, converse empirical findings indicate that church leaders would be able to gain a better understanding if they were prepared to become aware of the realities of young people’s lives and are willing to participate and assist young people instead of ignoring and denying reality. Boonstra (2011) found that individuals exposed to appropriate information on sex and sexuality are inclined to postpone their sexual activities. According to the World Council of Churches (2002), it is essential that churchgoers receive supportive, protective pastors in the churches as well as complete, accurate and updated information on how to remain healthy in a context of HIV.

### Theme 5: Religion and Condom-Related Beliefs

Students’ discussions revealed that religious leaders conveyed negative and confusing messages concerning condom use.

A Catholic male referred to the confusion relayed to youth, as follows:

…*Like in the Catholic Church, you’re told not to use a condom. This also confuses young people who want to act responsible*- *but now they remember what they hear in church. If I want to have sex now (they say) don’t use a condom*… *so now what*… *Who waits for marriage?*

A Christian female stated.

*We are not supposed to be having sex* (outside of marriage)… *that’s what they* (religious leaders) *tell you*… *but we all know what’s really happening*… *so not using a condom is stupid with all the diseases you can get*… (Other students agree).

Based on the student responses, it can be deduced that students in this study are sometimes confused by some of the messages received from religious leaders concerning the use of condoms. Students felt that religious leaders did not evaluate the meaning of their messages and its negative implication for young peoples’ sexual health as they chose to ignore the reality of individuals engaging in sex outside of marriage.

In line with the above finding, research (Kardas-Nelson 2009) indicates that some religious institutions have to re-examine the norms that they apply particularly to the use of condoms, given the high number of HIV infections in Southern Africa. One pertinent example was the request made by The South African Catholic Bishop’s Conference to the Vatican to review the stance on condoms as a preventive measure, particularly in situations where one person in a marriage is HIV positive, and the other is HIV negative (Kardas-Nelson 2009). Another reason why some religious institutions oppose condom use is the fear it will encourage promiscuity (Aguwa 2010; Foster et al. 2009; Krakauer and Newbery 2007). Oluduro (2010) cautions about the dangers of churches that continually provide misinformation on condom use: the use of condoms is portrayed as a direct, immoral and incorrect tool against HIV and AIDS. Oluduro (2010) further advises that churches rather remain silent or refrain from providing negative and incorrect information on the use of condoms.

### Theme 6: Perceptions of Religious Leaders as Role Models

Oluduro (2010) asserts that religious leaders are perceived by their communities as respected members of their society. Their attitudes and actions are examples of good moral conduct. Furthermore, their closeness to their communities allows them to impact on community behaviour. Therefore, any messages, even conduct related to morality and HIV and AIDS, displayed by these religious leaders are considered as shaping the attitudes and the behavioural patterns of their followers (in the community) about the epidemic.

In the current study, the immoral actions of some religious leaders, who should be regarded as role models, create confusion amongst the community and the participants. One participant stated the following:

*This sometimes confuses you, you don’t want to do these things (have sex) then these people who are your religious leaders are doing it. They’re also sometimes are HIV positive themselves and they’re not*… *how you say… good role models. They are confusing (a person). They must try to live by example*… (Other group members agree).

It can therefore be inferred that students display scepticism and confusion concerning the moral conduct of some religious leaders. Empirical evidence substantiates these views. Keikelame et al. (2010: 67) suggest that religious leaders who are promiscuous may be so because “fear may be especially potent among clerics and other religious leaders who are themselves HIV-positive”. They further note that religious leaders could by way of example play an important role in reducing stigma associated with being tested for HIV. This could be accomplished through their own public participation in voluntary counselling and testing (VCT) programmes.

### Theme 7: Emerging Adults General Concern for the Moral Decay of Society

Participant sentiments concerning the decline of societal morals and values are expressed in the following excerpt.

A Christian female student’s comment sums this up:

*I think it’s become more okay for them* (youth) *to do this type of thing, have unprotected sex, do drugs. Before*, (previous generations) *if someone was seen pregnant it was a shame. Nowadays they’re girls walking around flashing their stomachs and they’re about 15 or 16* *years old. It’s become more acceptable to have sex and to show it off to everyone. There’s no more shame*

A male Muslim student added

*Things have changed…The imams and church leaders should not only preach but, become involved in checking on* (monitoring content) *what we* (young people) *see on TV… and even in songs … sex is viewed in this country every day, its everywhere in your face … part of life.*

From the participant responses, it can be deduced that participants expressed concern for the decay in the moral fibre of society and that religious institutions should take an active stand in assisting youth with youth-specific challenges. Participants agreed that changes in society and rapid global change as well as advancement in technology are the cause of disappearing traditional morals and values. They feel that there is a decline in morals and values as society seems to tolerate moral decay more readily. They also appeal to religious leaders to be the moral custodians of society by censoring media and what young people are exposed to regularly.

Beckmann (2009) and Van Dijk (2009) concur with these findings and indicate that for various religious groupings, young people have expressed views that the HIV predictors are indicative of social moral decay. This is epitomised in society’s general acceptance and slackening of gender roles, sexuality and sexual mores, such as acceptance of prostitution and homosexuality.

## Theme 8: Perceptions of Whether Religion has an Influence on Young People’s Sexual Decision-Making and Practices is Highlighted in the Following

Participants in this study believed that religion was not a strong enough factor to prevent sexual activity from occurring. Some students indicated that having strong religious backgrounds did not necessarily influence their choice to engage in sexual activity. The following excerpt substantiates this:

*It’s so difficult*…(to abstain even with strong religious beliefs) *I have a friend*… *who*…*is religious,…she’s a reborn Christian,*… *She’s been dating… a guy studying for a captain, for a year. And they’ve had sex. He’s been away for*…*months, he’s back for a month*… *So I said to her, What are you gonna do?* (have sex*) because you’re not allowed to do this (have sex) anymore?*…*Everyday she’s having it. I’m like where’s your self*-*control?* - *You see, once people start doing it they don’t have the self*-*control to stop themselves, they just go for it* (have sex).

Another participant added:

*It* (religion) *used to be important not anymore*…*People ignore religion. People nowadays don’t take religion seriously. There’s no religion that will keep you from having sex, you feel nothing.* (No sense of morality)… *they’ve given up.* (Religious institutions have given up). (Group agrees).

*I come from a home where both my parents are committed Christians, my father’s a deacon in the church my mother’s on the church board. They drill you with rules all the time. You can’t have sex,. Now that’s what makes you rebel because when I was younger she wouldn’t even want me to dance with my male cousin. She would force me off the dance floor because dancing was bad. Once you rebel you do many things. Like when I was 15 I used to go clubbing. My parents didn’t even know this. That was the best part and I used to come in 2,3 in the morning with my brothers and they didn’t know.*

A Muslim male student added:

*At first my religion had a influence on me*… *in some way*… *for me not having sex*…

.(laughs, uncomfortably)… *But afterwards*… *I had sex*…*I’m still having sex*… *and I know this is wrong*…

Participants indicated that decision-making depends on the individual. This is summed up in the following extract by a Hindu student:

*It depends on you and the decisions you make and the relationship you have with God.You are responsible for your own actions*… *if you want to do it* (have sex),… *do it… but take responsibility*… (Others in the group agree).

These findings reflect views consistent with the emerging adult developmental phase. Participants’ scepticism concerning the role of religion as an influence on their sexual practices may be characteristic of a broader scepticism of social structures during the emerging adult developmental phase. A re-examination of faith and beliefs is a central part of emerging adulthood (Shuster and Mongetta 2009). However, during this period, emerging adults are less likely to be involved in religious institutions and detach themselves from the faith of their childhood and family background (Arnett and Jensen 2002; Barry and Nelson 2008).

According to Barry and Nelson (2005), emerging adulthood is best characterised as a period during which young people question the beliefs in which they were raised, place greater emphasis on individual spirituality than association with a religious institution and select the aspects of religion and spirituality that best suit them Arnett and Jensen (2002) found that individuals placed great emphasis on critical thoughts about spiritual issues rather than accepting an existing belief completely. Researchers on religion and youth trends concur that young people tend to rebel against the general, undefined spirituality of their parents and are in search of environments with more defined rules that assist them in coping with everyday problems (Zoll 2004).

Thus, the typical features of the emerging adult developmental phase (Arnett 2000) and Beaudoin’s views (1998) of Generation X, which reflects scepticism and the need to question religious institutions, seem to support the secularisation hypothesis. Similarly, students in this study, agree that religion has no influence on their daily life decisions to engage in sexual behaviour (multiple partners, unprotected; sex without condoms). Furthermore, modern society’s uncertainty in religion is apparent as religion seems to have limited influence on the manner in which society operates concerning its morals and values.

### Discussion of the Findings in Relation to Secularisation Theory

The findings of this study as reflected in the eight themes support the secularisation hypothesis in terms of the three levels of analysis as identified by Glasner (1977). These levels are: (1) interpersonal—religious beliefs do not guide individual behaviour/attitudes, (2) organisational—the church/religious institution is no longer important and (3) cultural—society is not influenced by religious ideas. Glasner further states that the possibility exists that secularisation could occur at one level but not necessarily at others.

In this study, emerging adult student responses infer that secularisation may have occurred on all three levels in relation to religious influences on sexual decision-making and sexual behaviours. Participants state that religion has no effect on their sexual decision-making (relating to level one of Glasner’s analysis) and that religious organisations are becoming defunct (relating to level two of Glasner’s analysis) as they no longer influence society (relating to level three of Glasner’s analysis). This is further supported in a study by Farmer et al. (2009) where a high rate of sexual behaviour was reported by individuals from all religious affiliations in their sample. This indicated a general lack of religious regulation of sexuality which also supported the secularisation hypothesis that religious influence is on the decline. In line with van den Toren (2003), Martin (2011) and Pickel (2011), cognisance should be taken of the various cultural and societal factors that influence the emerging adult student population in this study. Consequently, cultural and societal variations could thus influence the interpretation of secularisation. As mentioned, students come from diverse cultural and religious groupings, within the South African society. South Africa is a new democracy, with strong human rights having the first democratic elections in 1994. Furthermore, South Africa is a mix of modern and third world and considered an emerging economy with globalisation playing a key role. The influence of modern technology is very evident. Furthermore, the vast majority of students in this study (and their families) form part of the urbanisation process in their move from rural areas to cities with Cape Town experiencing an influx of rural students residing in squatter camps in and around the city. These students also form part of the poorer members of society in terms of economic status. Moreover, secularisation has been discussed and evaluated as a Western societal concept, while this study is based on the findings of emerging adult students’ views and perceptions of the influence of religion on their sexual practices in an (African) South African context.

Despite these dilemmas, secularisation theory still appears to be “a useful and meaningful analytical construct” (Yamane 1997: 109; Pickel 2011), which assists in analysing the development of the various kinds of religious beliefs. The researcher acknowledges that secularisation theory is not without fault, but concurs with Pickel (2011) who indicates that though secularisation is a complex process rooted in highly diverse analytical settings of social and political change, it is able to evoke numerous varied effects on the social and individual level. Pickel (2011) further advises the use of a conceptualisation of a *contextualised* or *context*-*sensitive* secularisation theory. He asserts that secularisation as a process is not universal, but context dependent, and therefore often nonlinear. Secularisation (as well as the revitalisation of religion) is mainly a product of circumstance—this is aligned with the assumptions of secularisation theory as used in this study.

## Conclusion

This study’s findings are consistent with secularisation theory about religion’s diminishing influence on society with specific reference to sexual decision-making and sexual behaviours amongst emerging adults. However, participants in this study provide valuable recommendations about how religion could become relevant in assisting with the current social issues faced by young people, particularly with regard to the promotion of responsible sexual decision-making and behaviours. These recommendations are evident in the findings as discussed under the aforementioned eight themes.

## Limitations

The findings of this study could have been influenced by cultural perceptions in relation to gender. As the researcher of this current study is female, this factor could have influenced the sharing of information by participants, relating to sexual practices and religion. Cultural and religious norms for some individuals could deem it inappropriate for males to discuss sexual issues with a female particularly in relation to religion. Thus, the ease of eliciting information from particularly male students could have been influenced by this factor. Furthermore, because of the sensitive nature of the topic (religion and sexual practices) it was probable that some students might have been under more pressure than others to respond in a socially acceptable manner to questions relating to sex. Furthermore, the possibility of reporting bias was possible because of self-reporting measures. Since sexual behaviour is confidential, the respondents could have under or over reported. But, studies in the USA oppose this view as researchers did not find that more religious individuals are more likely than others to respond in socially desirable ways (Regnerus and Uecker 2007).

Despite these shortcomings, the current study was able to explore the views and perceptions of a group of South African FET emerging adult college students regarding their sexual practices in relation to religion. Findings further suggest that, despite evidence of changes in sexuality and religiosity during emerging adulthood, these domains remain interrelated during this developmental period. Furthermore, these findings support the secularisation theory hypothesis that predicts the decline of religious involvement in modern secular life.

Given that South Africa is a multicultural society, paucity exists concerning information regarding religion and sexual behaviour particularly in the emerging adult student cohort. Future studies should focus on this emerging adult student population and explore these various dimensions of religiosity and sexuality in the various cultural and religious groupings present in this country and investigate how religiosity and sexuality predict each other over time.
